# Aqueous mechano-bactericidal action of acicular aragonite crystals

**DOI:** 10.1038/s41598-021-98797-w

**Published:** 2021-09-28

**Authors:** Nobuaki Negishi, Tomohiro Inaba, Yukari Miyazaki, Genki Ishii, Yingnan Yang, Setsuko Koura

**Affiliations:** 1 Environmental Management Research Institute, National Institute of Advanced Industrial Science and Technology (AIST), 16-1 Onogawa, Tsukuba, 305-8569 Japan; 2 Department of Applied Chemistry, Chiba Institute of Technology, 2-17-1 Tsudanuma, Narashino, 275-0016 Japan; 3 Graduate School of Life and Environmental Science, University of Tsukuba, 1-1-1 Tennodai, Tsukuba, Ibaraki 305-8572 Japan

**Keywords:** Environmental, health and safety issues, Photocatalysis

## Abstract

Nanoneedle structures on dragonfly and cicada wing surfaces or black silicon nanoneedles demonstrate antibacterial phenomena, namely mechano-bactericidal action. These air-exposed, mechano-bactericidal surfaces serve to destroy adherent bacteria, but their bactericidal action in the water is no precedent to report. Calcium carbonate easily accumulates on solid surfaces during long-term exposure to hard water. We expect that aragonite nanoneedles, in particular, which grow on TiO_2_ during the photocatalytic treatment of calcium-rich groundwater, exhibit mechano-bactericidal action against bacteria in water. Here, we showed that acicular aragonite modified on TiO_2_ ceramics prepared from calcium bicarbonate in mineral water by photocatalysis exhibits mechanical bactericidal activity against *E. coli* in water. Unmodified, calcite-modified and aragonite-modified TiO_2_ ceramics were exposed to water containing *E. coli* (in a petri dish), and their bactericidal action over time was investigated under static and agitated conditions. The surfaces of the materials were observed by scanning electron microscopy, and the live/dead bacterial cells were observed by confocal laser scanning microscopy. As a result, the synergistic bactericidal performance achieved by mechano-bactericidal action and photocatalysis was demonstrated. Aragonite itself has a high biological affinity for the human body different from the other whisker-sharpen nanomaterials, therefore, the mechano-bactericidal action of acicular aragonite in water is expected to inform the development of safe water purification systems for use in developing countries.

## Introduction

It was recently revealed that the surfaces of dragonflies, cicada wings, and the skins of gecko’s feature nanoneedle structures that show antibacterial activity^1–6^. Reported artificial reproductions of these biomimetic structures, demonstrate similar antibacterial activity, known as the mechano-bactericidal effect^7–17^. Studies are steadily elucidating these mechano-bactericidal mechanisms. The main consequences of mechano-bactericidal actions are the sterilization of solid surfaces and the inhibition of biofilm formation. A significant advantage of the mechano-bactericidal mechanism is the absence of chemical reagents; this property of nanoneedle biomimetic structures is garnering the attention of scientists for their potential to feature in environmentally friendly and sustainable bactericidal technologies.

Natural water typically contains various mineral components. Many are familiar with the white precipitate (scale) that forms and adheres to faucets; it develops when the concentrations of certain mineral components in tap water are relatively high. This white precipitate is calcium carbonate, with the crystal structure of mainly aragonite or calcite; significantly, aragonite may have a needle crystal habit^18–20^. In a previous study, we found that calcium carbonate forms on the surface of TiO_2_ photocatalysts during the photocatalysis of calcium bicarbonate contained water, moreover, the calcium carbonate mainly demonstrated the crystallinity of aragonite with nano- to micrometer-sized needles^21^. We expected that these aragonite nanoneedles would continuously kill any bacteria in flowing water upon contact through the mechano-bactericidal action associated with their topography. As mentioned above, the most famous mechano-bactericidal efficiency is the sterilization of bacteria adhered to the solid surface, and almost unknown the elimination of bacteria in flowing water by mechano-bactericidal effect. It would be a new discovery if the aragonite needle crystal has a mechano-bactericidal effect in the water. The mechano-bactericidal action of acicular aragonite in water will be expected to inform the development of water purification systems for use in developing countries in which countries have a problem with safe water access. One of the reasons is that calcium carbonate itself is a non-toxic compound, in addition, this is because calcium bicarbonate, which is a raw material for calcium carbonate, is relatively universally contained in groundwater. That is to say, it is also expected that the calcium bicarbonate in groundwater will use to photocatalytic repair (i.e., self-repair function) the needle aragonite with damage due to long-term use. Therefore, we studied the mechano-bactericidal action of aragonite nanoneedles against Escherichia coli in the water phase.

## Results and discussion

### Growth of aragonite and calcite on TiO_2_ ceramic surfaces

**Figure 1:**
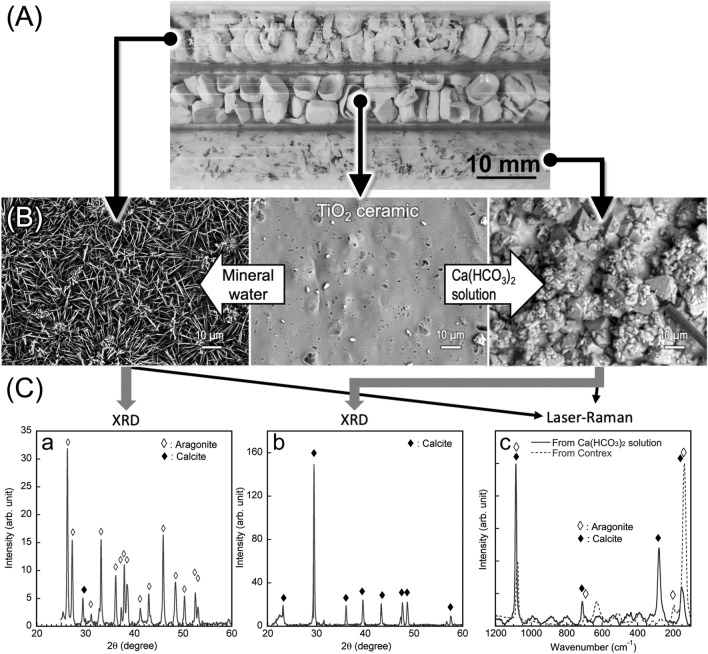
Accumulation states of CaCO_3_ onto TiO_2_ ceramics. (**A**) Image of the states after mineral water circulation under UV irradiation. Top: Contrex circulated system. Middle: original TiO_2_ ceramic. Bottom: Ca(HCO_3_)_2_ solution circulated system. (**B**) SEM images of modified TiO_2_ ceramic surfaces by CaCO_3_. Left: TiO_2_ modified during Contrex circulation. Center: original TiO_2_ ceramic. Right: TiO_2_ modified during Ca(HCO_3_)_2_ solution circulation. (**C**) XRD spectra of crystals precipitated from Contrex (a) and Ca(HCO_3_)_2_ solution (b), and Laser-Raman spectra of crystals precipitated from Contrex and Ca(HCO_3_)_2_ solution (c).

Calcium carbonate accumulates on the TiO_2_ ceramic surface, as shown in Fig. 1, during the long-term circulation of mineral water containing calcium bicarbonate under UV irradiation (Fig. 1A). Mineral water containing only calcium bicarbonate produced a precipitate characterized by hexagonal crystal (including various large and small disphenoids and 8-faced scalenohedron crystals) (Fig. 1B right), while mineral water (such as Evian and Contrex) containing not only calcium bicarbonate but also magnesium and strontium ions produces a precipitate characterized by orthorhombic acicular crystals (Fig. 1B left). As shown in Fig. 1C, the X-ray diffraction (XRD) (a and b) and laser-Raman spectra (c), respectively, reveal that the crystal structures of the hexagonal and orthorhombic acicular calcium carbonate are calcite and aragonite, respectively.

Acicular aragonite crystals did not form on the TiO_2_ ceramic surface when mineral water was circulated over the photocatalyst in the absence of UV irradiation. The lengths of the acicular crystals ranged from 10 nm to a few micrometers, depending on the duration of circulation and the concentration of calcium bicarbonate in the mineral water. It is expected that the large (micrometer) acicular crystals will capture the bacteria in a water-flow system, while the small (sub-micrometer) acicular crystals exert a mechano-bactericidal effect.

### Mechano-bactericidal efficiency

**Figure 2:**
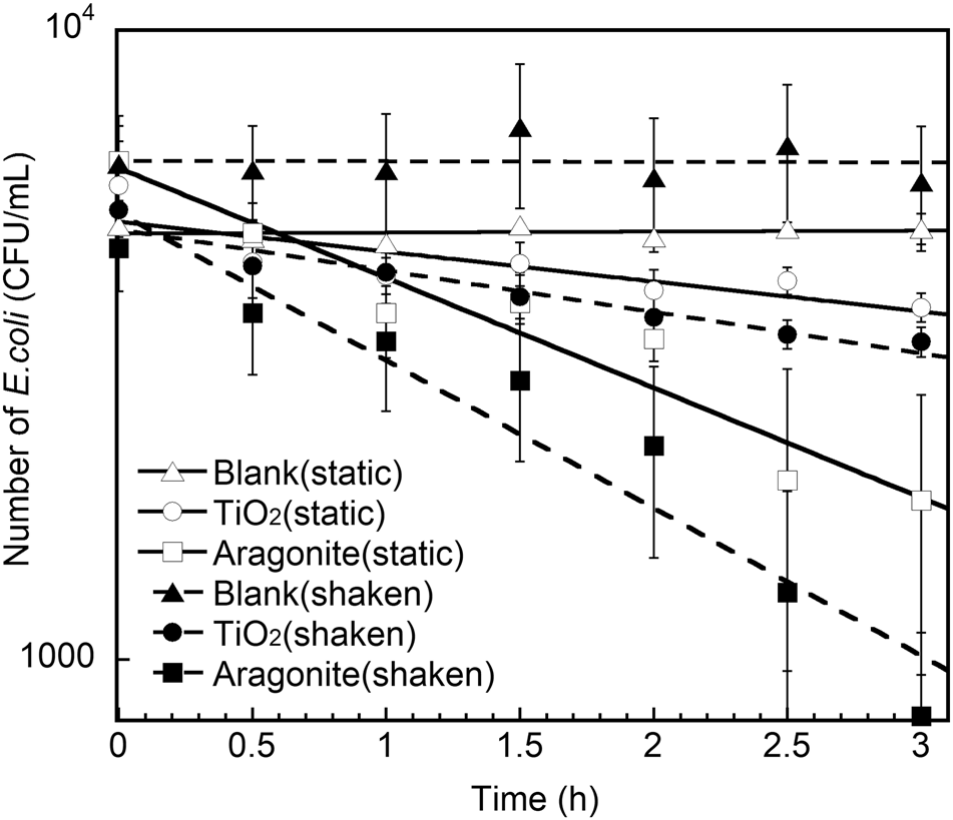
Over time changes in the number of underwater *E. coli* on unmodified and CaCO_3_-modified TiO_2_ ceramics under static and shaken conditions.

The changes in the number of *E. coli* cells in underwater over time are shown in Fig. 2. The number of *E. coli* cells did not change significantly in a saline water system. In a system containing unmodified TiO_2_ ceramics, the number of *E. coli* cells decreased slightly. In a system containing aragonite-modified TiO_2_, the number of *E. coli* cells decreased remarkably with or without shaken. The decrease in the number of bacterial cells under static conditions indicates that *E. coli* was captured by acicular aragonite owing to its own motor function, and the improvement in the antibacterial action achieved by agitation was less significant than the antibacterial action associated with the motor function of the bacteria.

**Figure 3:**
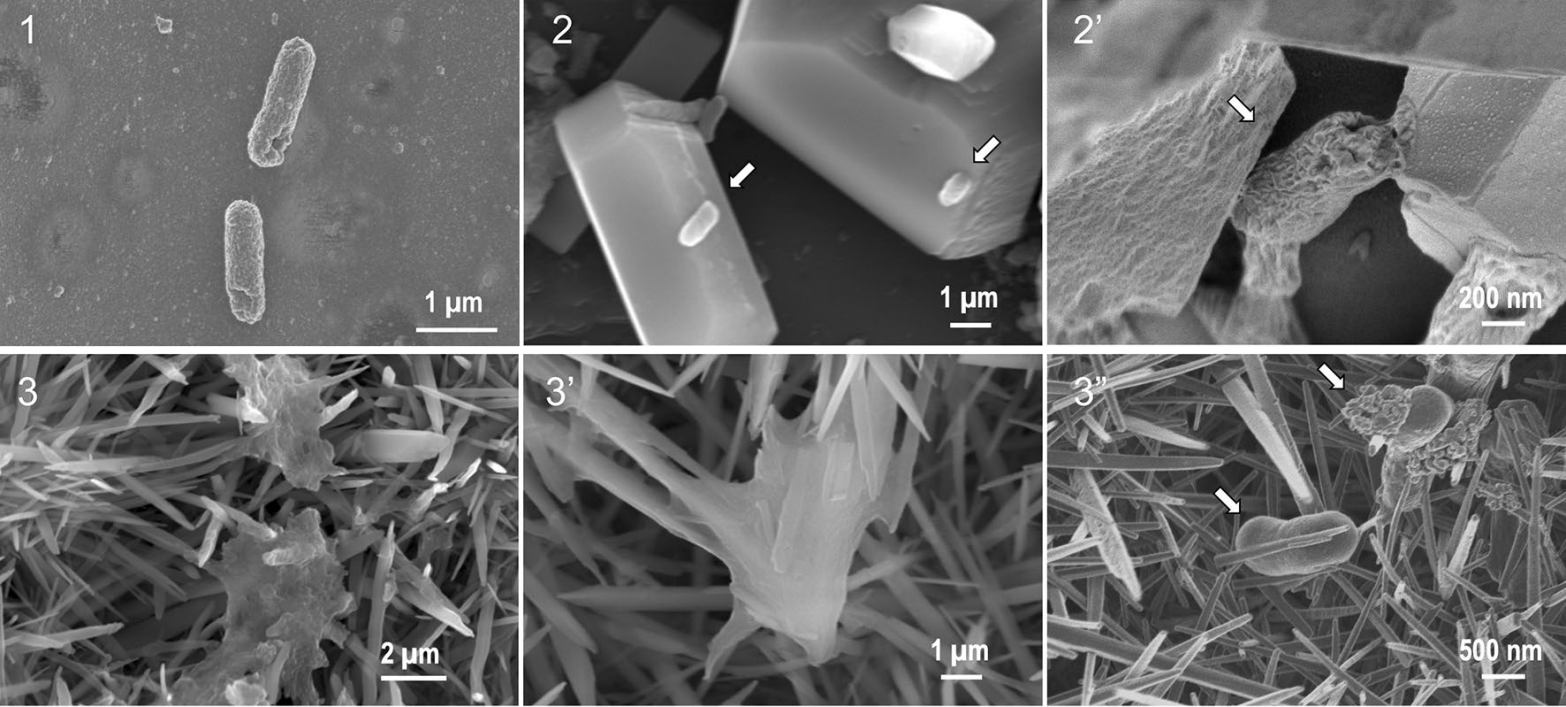
SEM image of *E. coli* on unmodified and modified TiO_2_ ceramic surface after 16 h immersed into *E.coli* contained water. TiO_2_ ceramic surfaces (1), Calcite-modified TiO_2_ ceramic surfaces under low (2) and high (2′) magnification, Aragonite-modified TiO_2_ ceramic surfaces under low (3), middle (3′), and high (3″) magnification.

Figure 3 display SEM images of the surfaces of unmodified, calcite-modified, and aragonite-modified TiO_2_ ceramics that were immersed in saline water containing *E. coli* for 16 h. The distinctive shapes of *E. coli* cells on the unmodified (Fig. 3 (1)) and calcite-modified TiO_2_ ceramic surfaces (Fig. 3 (2) and (2′)) did not change after 16 h. In contrast, the distinct shape changes of *E. coli* cells were observed on the aragonite-modified TiO_2_ ceramic surfaces. In Fig. 3 (3) and 3 (3′), the cell membrane was observed to be stretched and leathery. In Fig. 3 (3″), a situation was observed in which a substance that appeared to be protoplasm was ejected from the stab wounds of *E. coli*.

**Figure 4:**
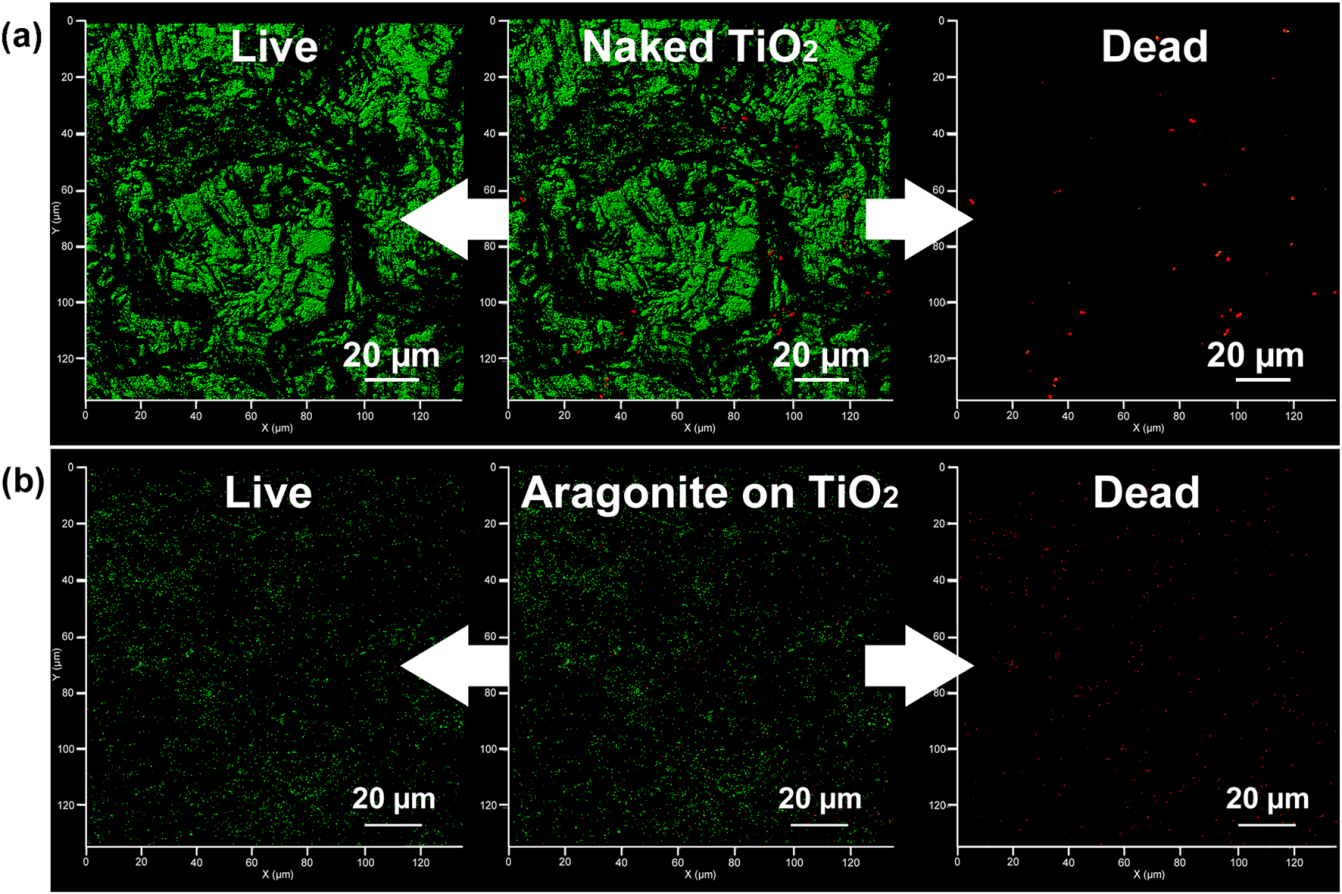
Confocal microscopy image of stained *E. coli* on an unmodified TiO_2_ ceramic surface (**a**) and an aragonite-modified TiO_2_ ceramic surface (**b**). The L/D composite image is shown in the center, and the distribution of live and dead bacteria is shown on the left and right, respectively.

Figure 4 show images of live/dead *E. coli* cells on unmodified TiO_2_ ceramic (a) and aragonite-modified TiO_2_ ceramics (b) obtained by confocal microscopy after 16 h in an *E. coli* solution under static condition (5 × 10^3^ CFU/mL). The green color indicates viable cells, and the red color indicates dead cells. The total number of cells (dead and alive) on the aragonite-modified TiO_2_ ceramic surface was much smaller than the number of cells on the unmodified TiO_2_ ceramic surface. The small number of dead cells observed suggests that the nucleic acid, which is stained with a fluorescent dye, eluted into the aqueous phase through the punctured cell wall (impaled by acicular aragonite: as shown in Fig. 3 (3″)). It is considered that this loss of nucleic acid (and fluorescent marker) accounts for the low incidence of cells (dead) observed by confocal microscopy on the aragonite-modified TiO_2_ ceramic surface.

**Figure 5:**
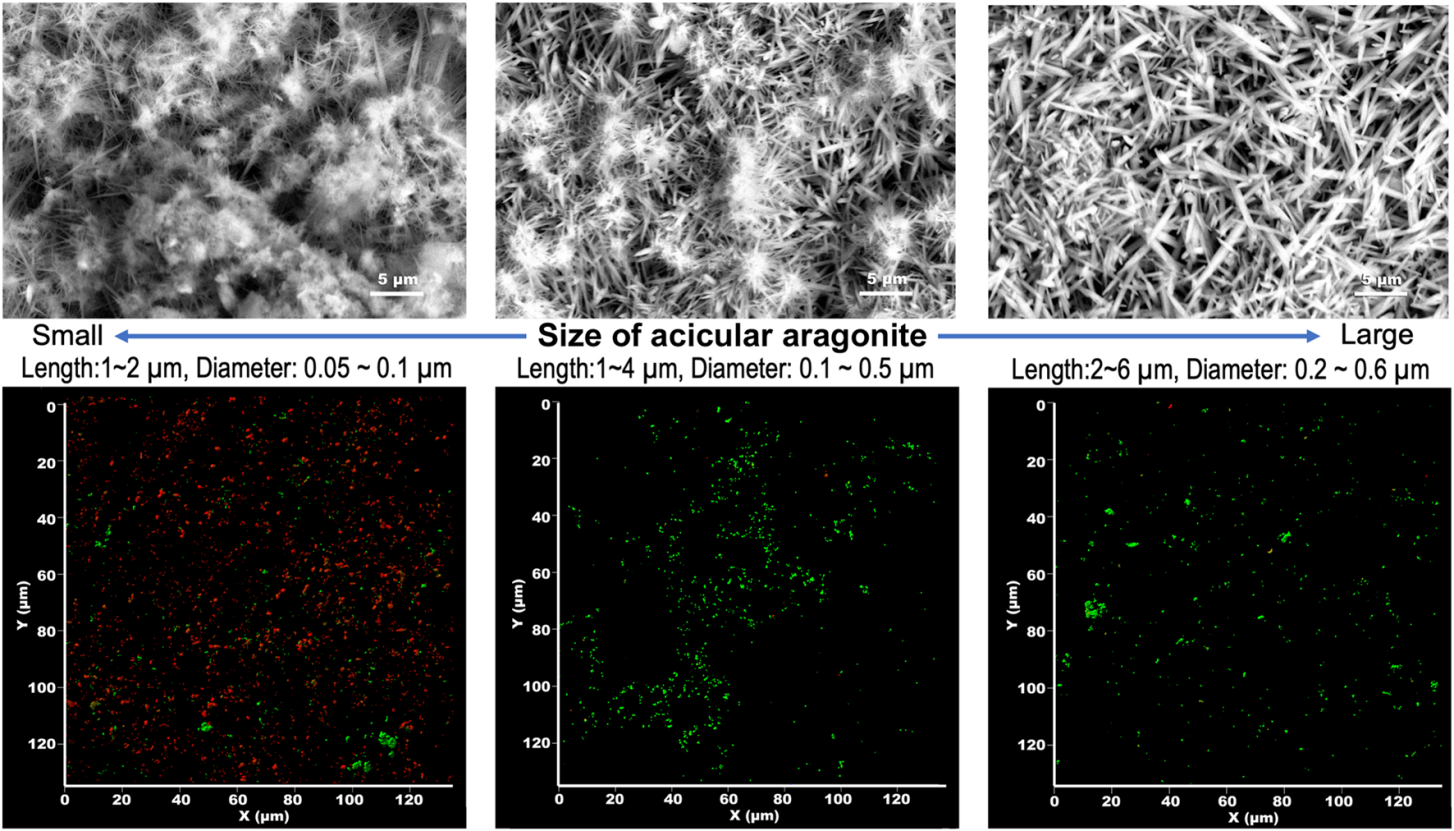
Relationship between the crystal size of acicular aragonite and bactericidal performance. Upper and Lower row shows the SEM image and the Confocal microscopy image of acicular aragonite-modified TiO_2_ ceramic surface with increase of aragonite preparation time, respectively.

The fate of bacteria captured by nanoneedle structures has already been reported. Hazel et al. and Jenkins et al. found that nanoneedles penetrate the cell membranes of bacteria^11,22^, resulting in sterilization. Wu et al. considered the relationship between the length of the nanoneedle that penetrates the bacterial-cell wall and the inter-needle distance^9^. They reported that the stretching of the cell membrane increases with the increasing density of the nanoneedles. Their experiment investigated the bacteria adhere to mechano-bactericidal solid surface; however, we expect that the bactericidal mechanism of us in water phase should be similar to them because we observed bacteria impaled on the acicular aragonite. We also investigated the mechano-bactericidal performance of acicular aragonite with different nanoneedle sizes. As shown in Fig. 5, the mechano-bactericidal performance of acicular aragonite was dependent on the size of the nanoneedles. This result is shown in Table 1. This needle-size dependence of mechano-bactericidal performance is consistent with the results of earlier studies for the solid surface^7,9,11,17,22^.

**Table 1:** The morphological characteristics of acicular aragonite and its L/D performance.

The size of acicular aragonite	Repeat time of circulation process	Acicular length (μm)	Acicular diameter (μm)	Dead (%)	Live (%)
Small	5	1–2	0.05–0.1	74.0	26.0
Middle	10	1–4	0.1–0.5	6.2	93.8
Large	15	2–6	0.2–0.6	6.5	93.5

**Figure 6:**
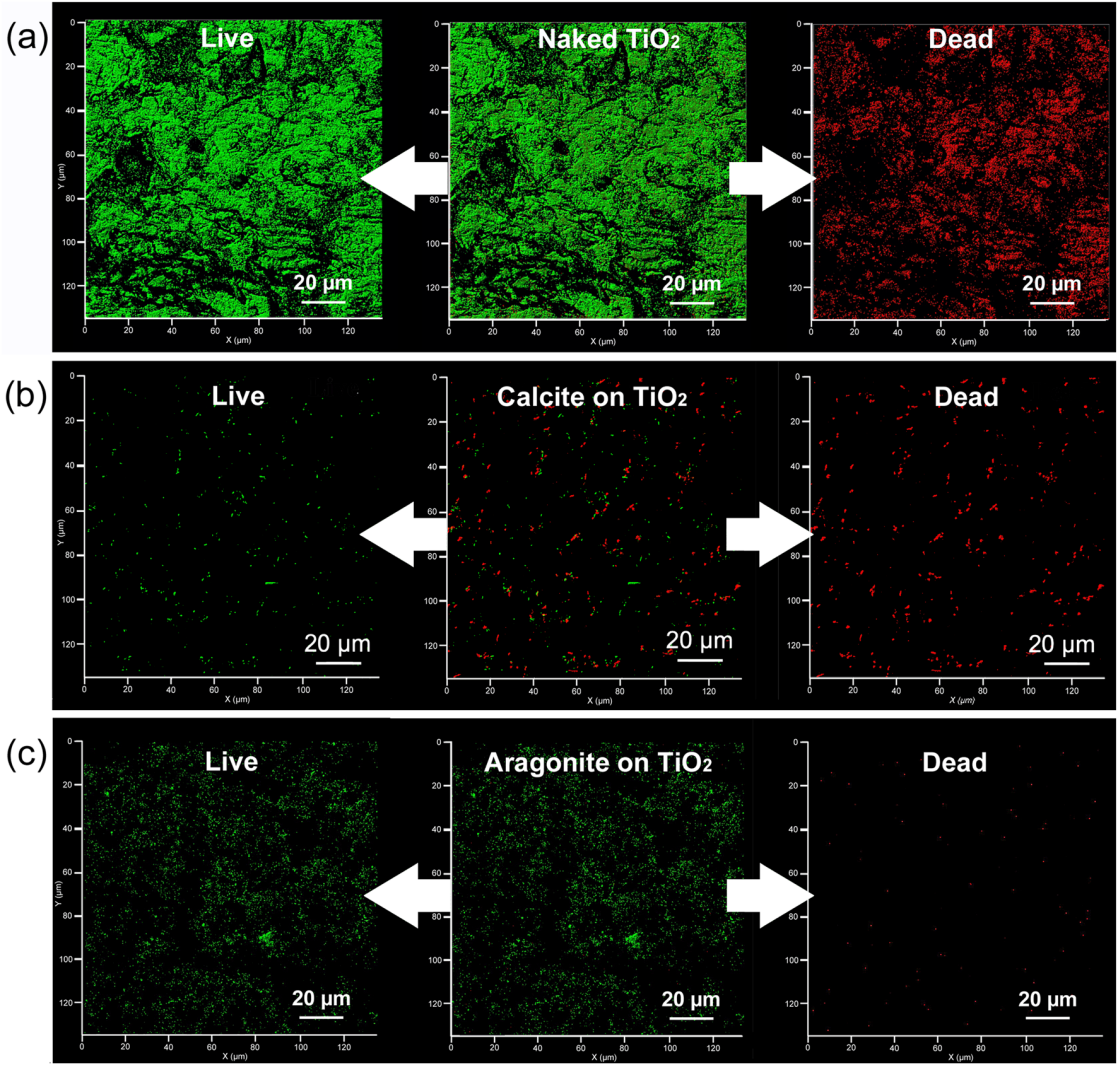
Observations of mechano-bactericidal effects in circulation systems. (**a**) Confocal microscopy images of stained *E. coli* on an unmodified TiO_2_ ceramic surface. (**b**) Confocal microscopy image of stained *E. coli* cells on a calcite-modified TiO_2_ ceramic surface. (**c**) Confocal microscopy image of stained *E. coli* on an aragonite-modified TiO_2_ ceramic surface. The L/D composite image is shown in the center, and the distribution of live and dead bacteria is shown on the left and right, respectively.

**Figure 7:**
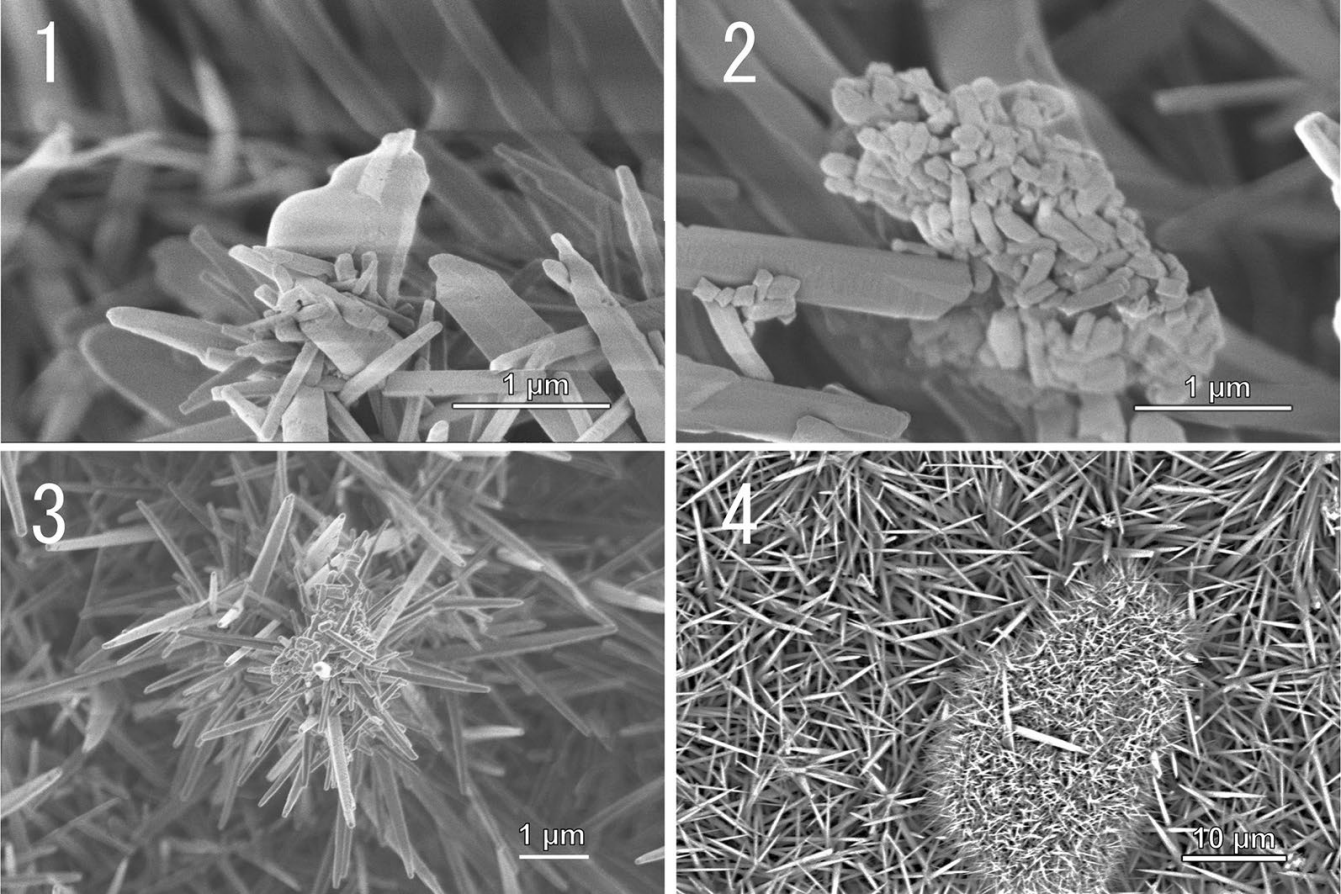
The SEM image of acicular aragonite-modified TiO_2_ ceramic surface after 3 weeks of exposure to circulating mineral water containing *E. coli*. A cell membrane fragment of a dead cell is observed and aragonite crystals have started to grow on its surface (1). Aragonite crystals start to grow and cover the surfaces of dead cells (2). The growth of aragonite crystals is stimulated by the circulating mineral water, resulting in the large acicular crystals observed (3). A cluster of dead bacterial cells covered by acicular aragonite (4).

Figure 6 reveals the mechano-bactericidal action in circulation systems. Unmodified, calcite-modified, and aragonite-modified TiO_2_ ceramics were packed into Pyrex glass tubes (300 mm × 10 mm (internal diameter)) and water containing *E. coli* (5 × 10^3^ CFU/mL) were circulated through these tubes at a rate of 50 mL/min. Figure 7a–c show images of the live/dead *E. coli* cells on the surfaces of the unmodified, calcite-modified, and aragonite-modified TiO_2_ ceramics, respectively, after circulating the aqueous phase for 3 h. A significant amount of viable *E. coli* cells was observed on the surface of the unmodified TiO_2_ ceramics (Fig. 6a). The total number of bacterial cells on the surface of the calcite-modified TiO_2_ ceramics (Fig. 6b) was less than that on the surface of the unmodified TiO_2_ ceramics; however, both viable and dead bacteria were observed. As shown in Fig. 6c, very few dead bacterial cells were observed on the surface of the aragonite-modified TiO_2_ ceramics, despite the presence of some viable bacteria; this result was consistent with the result obtained under static conditions (Fig. 4).

The most significant finding of this research is the underwater mechano-bactericidal action of acicular aragonite. Almost all of the reported nanoneedle, nanopillar, and whisker-shaped materials exhibit mechano-bactericidal action. However, it is known that asbestos, potassium titanate, carbon nanotubes, and metal nanowires demonstrate lung toxicity due to oxidative stress induced by their shape^23–25^. On the other hand, it is known that acicular aragonite does not show toxicity toward lung tissue, unlike the aforementioned nanoneedle, nanopillar, and whisker-shaped materials^18^. Aragonite, which is composed of calcium carbonate, easily dissolves in the living tissue and, as a result, morphology-induced toxicity does not manifest. In the course of water treatment through the mechano-bactericidal action using aragonite-modified TiO_2_ ceramics, we must assume the defluxion of nanoneedles into the water as a result of fracturing. Unlike body-soluble acicular aragonite, nanoneedle, nanopillar, and whisker-shaped nanomaterials are unsuitable for drinking water treatment because of the risk of fragment effluence.

We have already mentioned the possibility of damage to acicular aragonite during water treatment; however, it is also expected that the acicular aragonite will recover/self-repair using components in natural water (especially groundwater). More specifically, the self-replication ability of the system, and its mechano-bactericidal action, is anticipated. In fact, we found that acicular aragonite nucleates from dead *E. coli* cells during long-term circulation in Contrex system (Fig. 7).

### Combination performance of mechano-bactericidal effect and photocatalytic sterilization

**Figure 8:**
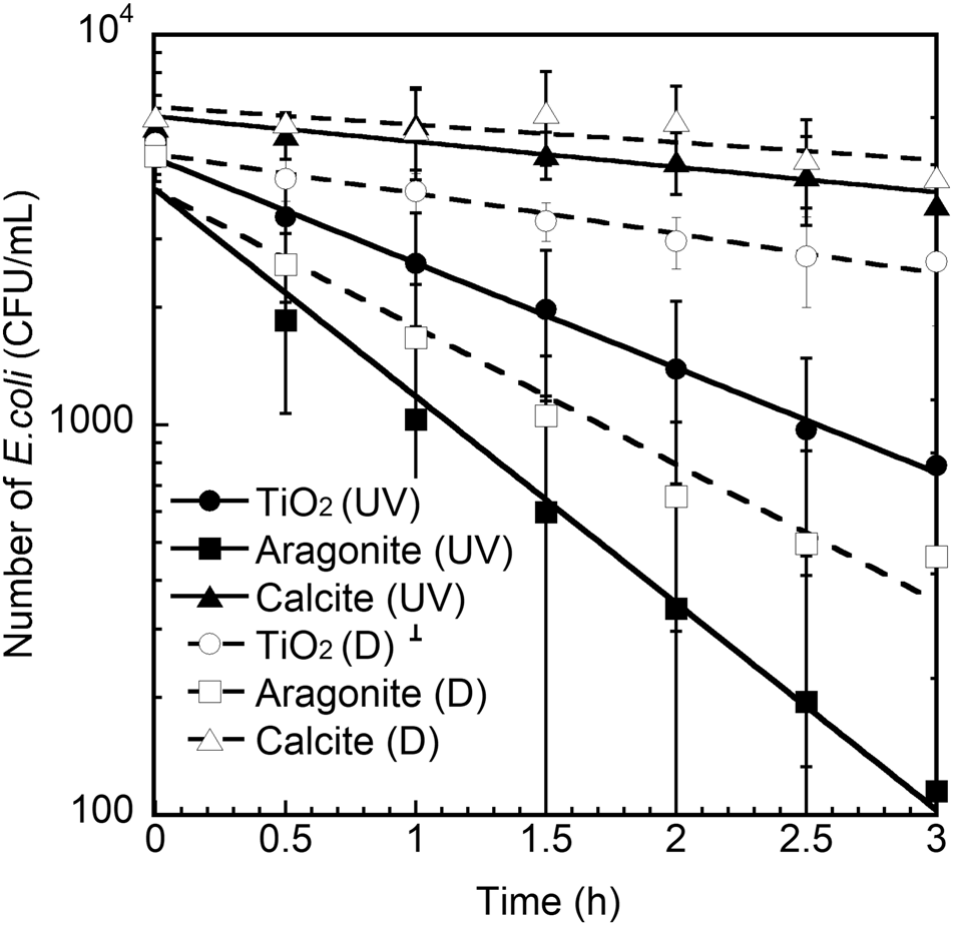
Change in the number of *E. coli* cells over time in circulation systems featuring unmodified, calcite-modified and agonite-modified TiO_2_ ceramics under dark (D) and UV light (UV) conditions.

Photocatalytic environmental purification can only be performed during the daytime and, for optimum performance, under sunny conditions^26–29^. However, ideal photocatalytic drinking-water purification systems for developing countries should achieve absolute performance under all weather conditions, not only sunny but also cloudy or rainy; possibly by combining photocatalytic activity and mechano-bactericidal action. Figure 8 shows the change in number of *E. coli* cells in circulation systems featuring tubes containing unmodified, aragonite-modified and calcite-modified TiO_2_ ceramics under dark and UV light conditions. The number of *E. coli* cells in the unmodified TiO_2_ ceramic system decreases significantly more under UV-A irradiation than dark conditions due to photocatalysis^30,31^. However, the number of *E. coli* cells in the aragonite-modified TiO_2_ ceramic system under dark conditions is lower than that in the unmodified TiO_2_ ceramic system under UV-A irradiation. The point to note is that the absolute rate of *E. coli* cell reduction in the aragonite-modified TiO_2_ ceramic system under UV-A irradiation (Sterilization rate const. = 1.22 h^−1^) was ~ 2.0 times of under dark (Reduction rate const. = 0.809 h^−1^), and ~ 3.0 times of the UV light conditions in the unmodified TiO_2_ ceramic system (Photocatalytic rate const. = 0.618 h^−1^) as shown in Table 2.

**Table 2:** Photocatalytic performance of unmodified, aragonite and calcite modified TiO_2_ ceramics and their specific surface area.

Material	Dark (h^−1^)	UV (h^−1^)	Surface area (m^2^/g)
TiO_2_ ceramic	0.236	0.618	61.7
Acicular aragonite on TiO_2_ ceramic	0.809	1.220	48.6
Calcite on TiO_2_ ceramic	0.105	0.149	18.2

In a previous study, we revealed why the photocatalytic activity of TiO_2_ does not decrease with increasing aragonite accumulation on its surface^21^. The photocatalytic reaction mainly involves the formation and migration of active species, such as ⋅OH, by UV irradiation of the TiO_2_ photocatalyst. The lifetime of ⋅OH, of around 2.7 µs^32^, is considered sufficient to allow it to migrate into the aqueous phase through the layer of aragonite. The densification of the aragonite layer is expected to impede the migration of active species. In reality, the aragonite layer on the TiO_2_ surface is very porous and a few micrometers thick. This porosity facilitates the migration of the active species, generated by photocatalysis, to the surface of the aragonite layer. This is one of the reasons why the photocatalytic sterilization performance of the aragonite-modified TiO_2_ ceramic photocatalyst exceeds that of the unmodified TiO_2_ ceramic photocatalyst. Moreover, the mineralization of bacteria on the aragonite-modified TiO_2_ ceramic photocatalyst is promoted by photocatalysis; as a result, the surface of this material will maintain a clean condition unlike that of an aragonite system without photocatalytic materials. In this experiment, we also carried out using the calcite-modified TiO_2_ ceramic. However, photocatalytic sterilization could not be achieved not only in dark conditions but also in UV irradiation, as shown in Fig. 8. Calcite formed a large hexagonal crystal different from a case of acicular aragonite, and it is considered that this calcite covered the TiO_2_ surface and prevents the diffusion of active species generated by UV irradiation. The situation in which calcite plays a lid for the TiO_2_ ceramic with a large surface area can be also inferred from the specific surface area observation results in Table 2.

As described above, we demonstrated the mechano-bactericidal treatment of water by acicular aragonite. However, several aspects require elucidation, such as the relationship between the optimum needle size of aragonite or flow speed and bactericidal performance, acicular aragonite associated toxicity against live body except for lung toxicity, and the mechano-bactericidal action of acicular aragonite against other bacterial species. As previously reported, the TiO_2_ ceramic photocatalyst, which is used as a substrate for acicular aragonite growth, is extremely strong and does not deteriorate during long-term use. Therefore, it is expected that access to safe water can be achieved in many developing countries with systems that combine mechano-bactericidal action and photocatalysis, such as the proposed acicular aragonite-modified TiO_2_ ceramic system, that obviate the use of disinfectants and concomitant chemical risks and high running costs.

## Conclusion

Acicular aragonite nano-needle, a metastable phase of calcium carbonate, precipitated on the photocatalytic surface by the photocatalytic reaction of calcium bicarbonate contained water such as ground water, showed a mechano-bactericidal effect in water which is not still known. Calcite, which is a stable phase of calcium carbonate, did not show any mechano-bactericidal effect. From the results of phase contrast microscopy, the aragonite phase showed a low density of dead bacteria on its surface, which may be due to the leakage of protoplasm to be stained into the water. Although the mechano-bactericidal effect alone was greater than the photocatalytic effect alone, the synergistic effect was observed for the aragonite modified photocatalyst, and the bactericidal rate in water was equal to the sum of the mechano-bactericidal and photocatalytic effects. The aragonite modified photocatalyst is expected to be used for drinking water purification in developing countries, the loss of aragonite needle crystal habit may occur due to its long-term use. However, it was confirmed that aragonite was precipitated from dead bacteria after long-term use of the aragonite modified photocatalyst in hard water, indicating that the material has a self-regenerating function to maintain its mechano-bactericidal effect and is a promising material for realizing safe water access in developing countries.

## Methods

### Substrates for CaCO_3_ growth

The preparation of a TiO_2_ ceramic photocatalyst employed as a substrate for the growth of CaCO_3_ crystals, such as aragonite, has previously been reported^21^. TiO_2_ is known as a photocatalytic material that has generally two crystal phases, anatase and rutile (sometimes brookite is also mixed). The photocatalytic activity of anatase is high, higher than that of rutile^33–35^. Unless specified otherwise, all of the experimental procedures were carried out in the absence of UV light; in particular, we used high temperature treated (750 °C) rutile TiO_2_ ceramic as a substrate for aragonite modification to reduce the risk of undesired photocatalytic reactions. On the other hand, anatase TiO_2_ ceramics calcined at 550 °C were used in the photocatalytic (under UV irradiation) experiments. This ceramic photocatalyst is generally more robust in water than other photocatalytic materials and, since its semipermanent use in the water is expected, very suitable for the treatment of drinking water in developing countries^36^.

### Growth of aragonite and calcite on TiO_2_ ceramic surfaces

Commercial mineral water is the most suitable starting material for the growth of acicular aragonite crystals on TiO_2_ ceramics. Certain brands of mineral waters are rich in calcium bicarbonate (as indicated by their ingredients labels of commercial products). While these mineral waters contain various minerals, only one precipitate, calcium carbonate, is generally formed. We used Contrex from France since this mineral water contains high concentrations of calcium bicarbonate and its pH is almost neutral. In contrast, the precipitation of a calcite reference sample required a pure calcium bicarbonate solution. The calcium bicarbonate solution employed in this study was the filtrate of a solution prepared by bubbling CO_2_ gas through a calcium hydroxide–saturated solution^37^. To grow aragonite (or calcite) on TiO_2_ ceramics, 500 mL of Contrex (or calcium bicarbonate solution) was introduced into a water flow line connected to a glass tube packed with TiO_2_ ceramics under UV irradiation. The circulatory system was the same as in our previous report^21^. After circulating for 8 h, the circulation system and content were dried under airflow for 16 h; this circulation and drying process was repeated until 15 times. Since the CaCO_3_ crystals grow larger by repeating this process, we obtained crystals of different sizes as shown in Fig. 5 and Table 1 by dividing the process into five steps. The crystalline structures of these precipitates were determined using X-ray diffraction (XRD; D2 Phaser, Bulker Germany) and laser Raman spectroscopy (NRS-4500, JASCO, Tokyo, Japan). The morphologies of the surfaces of these materials were observed by field emission scanning electron microscopy (FE-SEM; S-4700, Hitachi, Tokyo, Japan)^38^.

### Biological experiments

*Escherichia coli* K-12, purchased from NITE Biological Resource Center (NBRC No. 3301) as a model bacterium, was streak-cultured on an agar plate (NBNaCl medium: Bacto Peptone (Becton, Dickinson and Company, NJ, USA) 0.5%, beef extract (MP Biomedicals LLC, CA, USA) 0.3%, NaCl 0.5%, agar(FUJIFILM Wako Pure Chemical Corporation, Osaka, Japan) 1.5%), and then shake cultured at 60 rpm in liquid NBNaCl medium containing Bacto Peptone 0.5%, beef extract 0.3%, and NaCl 0.5% at 36 °C for 16 h*.* The *E. coli* growth curve was determined from the optical density at 600 nm measured with a cell density meter (CO8000, Biochrom, Cambridge, UK). The *E. coli* was cultured until the system reached a stationary state at 0.5–1 × 10^8^ CFU/mL. The cultured *E. coli* was diluted once with phosphate-buffered saline, and 0.1 mL of the diluted/undiluted culture was used to inoculate the NBNaCl culture media plates. The *E. coli* was cultured on the NBNaCl media at 35 °C for 24 h.

The number of colonies was counted using a colony counter and the average number of colonies was calculated. If the number of colonies per dilution factor differed by more than a factor of 2, the value for the culture medium with a lower dilution factor was used. All media, incubation flasks, micropipette tips, and filtration units (including bottle parts used for the preparation of bacterial suspensions) were sterilized by autoclaving at 121 °C for 20 min or by gamma sterilization.

### Fluorescent staining

SYTO9 is a membrane-permeable DNA staining reagent that stains the nuclear DNA of viable bacteria without membrane damage and emits 500 nm (green) fluorescence when exposed to light at a wavelength of 483 nm or lower. On the other hand, PI is not membrane-permeable, therefore it stains nuclear DNA of dead bacteria with membrane damage and emits fluorescence at 617 nm (red) when irradiated with light at a wavelength of 536 nm or lower. Based on these differences in fluorescence wavelengths, we determined the viability of *E. coli* adsorbed on the sample surface^39^. The final concentrations of SYTO9 and PI were prepared at 6 µmol/L and 30 µmol/L, respectively, and their mixed solution was used as the fluorescent staining solution (L/D reagent) in the viability determination. A drop of 0.1 mL of L/D reagent was added to the sample treated and the surface was covered, and the sample was allowed to stand for 15 min. The staining process was carried out in the dark to eliminate the effect of quenching of the fluorescent reagent.

### Fluorescence observation and image analysis

Observation of the fluorescent staining samples was performed by confocal laser microscope (LSM880; Carl Zeiss, Oberkochen, Germany). Observation was performed while the samples were immersed in sterilized ion-exchange water to eliminate the possibility of *E. coli* being killed by drying. During the observation, the fluorescence emitted by *E. coli* adsorbed on the sample surface was captured by irradiating the sample with a laser of a specific wavelength. From the images obtained by combining green and red fluorescence, we determined the viability of *E. coli*. The percentage of L/D was analyzed by ImageJ image analysis software. The green and red areas of the obtained images were split using the "Split Channels" function, and the percentage of alive or dead was calculated from the total number of pixels.

### Mechano-bactericidal experiment (incl. photocatalysis)

To analyze the mechano-bactericidal action of the materials, we performed one type of static and two types of dynamic experiments. In the static experiment, cultured *E. coli* (1 × 10^7^–10^8^ CFU/mL) solution was fed into culture tubes (volume: 5 mL), containing one piece of unmodified TiO_2_ ceramic, calcite-modified TiO_2_ ceramic, or aragonite-modified TiO_2_ ceramic, that was then left at rest at 4 °C for 16 h. The *E. coli* cells on the surface of each substrate were observed by confocal microscopy, after staining the cells with SYTO9 and PI, to estimate the live/dead bacterial cell abundance and evaluate their membrane integrity. In addition, the *E. coli* cells on the surfaces were observed by FE-SEM after fixation with glutaraldehyde^40,41^.

The first dynamic experiment was a time–course experiment; 50 mL of a solution containing *E. coli* (~ 5000 CFU/mL) was poured into petri dishes containing unmodified or aragonite-modified TiO_2_ ceramics and sampling was performed every 30 min for 3 h at 25 ºC under static and agitated conditions in a shaking incubator. For reference, this procedure was repeated without any substrates (blank). The initial number of E. coli cells in the respective samples, were differed between confocal microscopy and culture method. This differences are attributed to the different of the detection limits of confocal microscopy associated with these culture methods.

In the second dynamic experiment, to simulate a practical application, we circulated 250 mL of saline water (0.5 g/L) containing *E. coli* (5000 CFU/mL) through a glass tube packed with aragonite-modified TiO_2_ ceramics for 3 h at a flow rate of 50 mL/min. We also used the black-light blue fluorescent lamp (15W-BLB) for photocatalytic reaction as the light source when the photocatalytic experiment was carried out, and the UV-A (λ = 365 nm) intensity was fixed at 2 mW/cm^2^ at the position of the photocatalyst-packed tube. The UV-A intensity was measured by means of a UV power meter C9356-1 (Hamamatsu Photonics, Hamamatsu, Japan).

The solution was sampled every 30 min, and 0.1 mL of each sample was used to inoculate the NBNaCl culture media plates and cultured at 35 °C for 24 h. After each 3-h circulation, the apparatus was drained under air pressure and disinfected by circulating ethanol (FUJIFILM Wako Pure Chemical Corporation, Osaka, Japan) for two 30-min periods. Milli-Q water was passed through (not circulated) the apparatus for 2 h; the apparatus was again drained under air pressure before the aragonite-modified TiO_2_ ceramics in the apparatus was dried overnight under flowing air and UV irradiation (λ = 352 nm) to eliminate any residual organic compounds and bacteria on the surface of the TiO_2_ by photocatalysis. After the circulation experiment, aragonite-modified TiO_2_ ceramic was abstracted from the glass tube and the bacteria were observed by FE-SEM after fixation with glutaraldehyde.
